# Proteolytic activity in the brain of alloxan diabetic rats: Presence of a proteolytic activator in the cerebral extract

**DOI:** 10.4103/0973-3930.44078

**Published:** 2008

**Authors:** Nayeemunnisa

**Affiliations:** Al Ameen College of Pharmacy, Hosur Road, Bangalore - 560 027, India

**Keywords:** Alloxan diabetes, brain-dysfunction, glucose metabolism, proteins, proteolysis

## Abstract

**BACKGROUND AND AIM:**

Diabetes mellitus is known to cause neurological disorders due to impaired glucose metabolism involving decreased utilization of glucose by the brain tissues. The mechanisms responsible for failure of glycemic regulation in type-2 diabetes leading to neurological impairment need to be thoroughly elucidated.

**MATERIALS AND METHODS:**

Type-2 diabetes was induced in albino rat models with alloxan monohydrate (40 mg/kg i.v.). Cerebral cortex and medulla oblongata were investigated 48 h after alloxan administration for the alterations in proteolytic activity.

**RESULTS:**

Diabetes caused an elevation (*P* < 0.001) of blood glucose and also proteolytic activity in the brain.

**CONCLUSION:**

Impaired glucose metabolism in the brain was the key factor which was responsible for the elevated (*P* < 0.001) proteolysis leading to brain dysfunction.

## Introduction

Diabetes is characterized by impaired carbohydrate metabolism and uncontrolled glucose levels and perturbation of glucose homoeostasis.[1,2] Since brain is affected by recurrent episodes of hypoglycemia and poor metabolic control, protein metabolism undergoes serious alterations during diabetes.[3] Diabetes-induced hyperglycemia enhances the extent of neurological disorders due to enzyme inactivation.[4] Activities of enzymes connected to glucose metabolism and neuronal activities have been studied as a function of diabetes.[5,6] The present study aimed at identifying the limiting molecular events for the specific lesion which can be correlated directly to diabetes. I have herein ventured to report the degradation of proteins by protease activity in the brain of alloxan diabetic albino rat models. Presence of a proteinase activator in the cerebral extract of diabetic rats has also been presented. Protective intervention of plant extract has been reported.

## Methodology

Institute bred immature albino Wistar rats ranging in weight from 245–260 g were used for the present study. These animals were housed in polypropylene cages at laboratory temperature (26 ± 2 °C) and fed standard pellet diet (Hindustan Lever Ltd. Bombay, India). Water was available *ad libitum*.

### Induction of diabetes

Diabetes was induced by an intravenous injection of a freshly prepared aqueous solution of alloxan monohydrate (40 mg/kg).

The study protocol was approved by the Institutional Animal Ethics Committee. The animals were divided into two groups (group 1: control rats; group 2: diabetic rats) of 11 each.

The animals were decapitated and the brains were quickly removed and washed in ice-cold saline. Different regions of the brain were separated with sterilized fine-bent forceps and scalpel, weighed in an electric balance in mammalian Ringer and immediately used for the determination of different biochemical parameters.

Glucose and protein levels were measured by the colorimetric method of Nelson and Somogyi as described by Oser[6] and Lowry method,[7] respectively.

### Proteolytic activity

Protease activity in the tissue homogenate of different brain regions was estimated using casein (4% in 50 mM sodium phosphate buffer, pH 7.5). The assay mixtures (2 ml) were incubated for 3 h at 40 °C, after which the reaction was stopped by the adding 3 ml of 5% trichloro acetic acid. The protease activity was measured colorimetrically[7] and was expressed as micro mols of tyrosine equivalents liberated/h/mg protein. Also, the effect of the inhibitor pepstatin (specific for protease cathepsin D) on cerebral protease was determined. Pepstatin was from Peptide Institute Inc., Osaka, Japan. Optical density was measured using DU_2_ Beckman Spectrophotometer.

Effects of *in vitro* administration of the aqueous cerebral extract (0.5 ml) from alloxan diabetic rats on the brain protease activity levels of normal animals were studied. It was noted from preliminary experiments that only the cerebral extract from the diabetic animal was capable of causing significant increase of the enzyme activity in the brain of normal animals and therefore, the effects of *in vitro* administration of the extracts from cerebellum, medulla and whole brain of diabetic animals were not studied.

The assay system, on addition of brain aqueous extract from normal animals constituted the controls. Preliminary experiments indicated negligible difference between the enzyme activity levels of controls prepared by adding the cerebral extract from diabetic animals to the assay system after terminating the enzyme reaction and those receiving the brain extract from normal animals. Hence, the latter were used as the experimental controls. The assay system with the aqueous cerebral extract from the diabetic rats was the experimental samples.

Statistical analysis of the data was done using student's *t* test. *P* < 0.05 was considered significant.

## Results and Discussion

The weights of the animal and brain exhibited insignificant decrease as a function of the disease [Table 1]. The blood sugar level demonstrated 226% elevation as a function of diabetes [Table 1].

**Table 1:** Changes in the blood glucose levels of albino rats as a function of alloxan diabetes

	Body wt. (g)	Brain wt. (g)	Blood glucose (mg/l00 ml)
Control rats	80 ± 2.3	1.8 ± 0.08	100 ± 3.2
Diabetic rats	75 ± 1.6	17 ± 0.6	326 ± 7.5
All rats	−6.2*	−5.0*	+226***

Values are mean ± SD of 26 observations, @:

* Not significant

*** *P* < 0.001

The protease activity increased (*P* < 0.01) in the cerebral cortex and medulla oblongata as a function of alloxan-induced diabetes suggesting the functioning of high protein hydrolytic activity and possible utilization of the products for metabolic processes which undergo substantial change due to diabetes-induced pathological manifestations [Table 1]. Earlier studies also indicated such changes as a decline in the protein content and its synthesis as a function of diabetes.[5]

**Figure 1:**
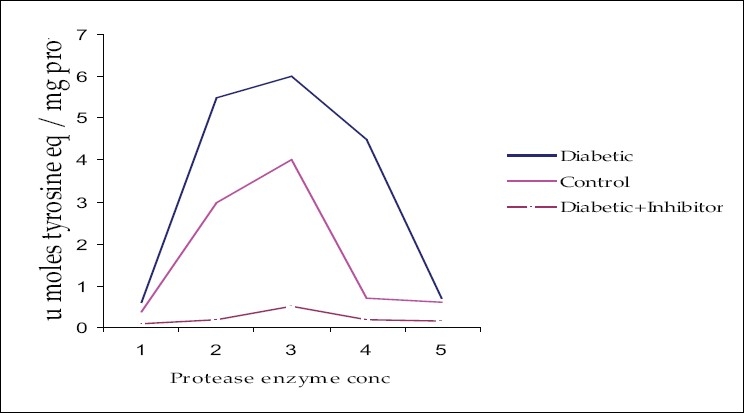
Inhibition of proteolytic activity by the inhibitor from the cerebral extract of normal animal

The use of the inhibitor pepstatin (specific for cathepsin D) categorized the proteolytic enzyme in the brain tissue to be cathepsin D [Figure 1].

On administration of cerebral extract from the brain of diabetic animals to the control tissue, the changes observed were those of diabetic manifestations indicating the presence of controlling factors which shift the neuronal functions paralleling the demands imposed by the diabetic conditions [Table 1].

It has been demonstrated that small bioactive peptides present in the CNS are implicated in neurotransmission and functional regulation. These processes are mediated through proteolytic enzymes.[8] Involvement of peptide hydrolases in the conversion and inactivation of specific neuropeptides emphasizes the enhanced proteolysis during diabetes leading to neuronal dysfunction.

Presence of a proteolysis activator in the brain cerebral extract of diabetic animals suggests the genesis of factor/s in the cerebral cortex of the brain during diabetes in albino rat models, capable of elevating the proteolysis of the brain tissue [Table 3]. This may lead to serious alterations in the dynamics of brain proteins and protein metabolism[5] leading to cellular impairments during diabetes [Tables 2 and 3 and Figure 1].

**Table 2:** Activity levels of protease in different regions of the brain of control and diabetic rats

	Cerebrum	Cerebellum	Optic lobes	Medulla oblongata
Control rats	47.4 ± 3.7	37.2 ± 5	14.0 ± 1.0	25.5 ± 2.2
Diabetic rats	58.1 ± 4.4**	48.2 ± 3**	15.8 ± 3.2***	35.4 ± 1.5**

Protease activity expressed as milli moles free amino acids/min/mg protein. Values are mean ± SD of 9 observations

** *P* < 0.01, between controls and diabetic

*** NS,Not significant

**Table 3:** Effect of cerebral extract on the protease activity in different brain regions of albino rats

Nature of the extract used	Cerebrum	Cerebellum	Medulla Ob
Normal cerebral extract			
Treated tissue (Control)	33 ± 4.2	38 ± 3.9	50 ± 2
Diabetic cerebral extract			
Treated tissue (Exptls)	69 ± 5.7	90 ± 4.4	71 ± 2.
Percentage Change	79.1**	93.47**	46.0*

Activity is expressed as micro moles of tyrosine equivalents/mg protein/hr, Values are Mean ± SD of 9 observations

* *P*<0.01

** *P* < 0.001

Activity levels of protease in the cerebrum, cerebellum, and medulla oblongata of normal animals determined in the presence of the cerebral extract from diabetic animals were significantly high [Table 3]. The changes produced by the nervous extract of diabetic rats indicated the presence of substances in the cerebral extract of alloxan diabetic rats capable of elevating protease activity of normal animal on *in vitro* administration.
